# The Grouping-Induced Numerosity Illusion Is Attention-Dependent

**DOI:** 10.3389/fnhum.2021.745188

**Published:** 2021-10-06

**Authors:** Antonella Pomè, Camilla Caponi, David C. Burr

**Affiliations:** ^1^Department of Neuroscience, Psychology, Pharmacology, and Child Health, University of Florence, Florence, Italy; ^2^School of Psychology, University of Sydney, Sydney, NSW, Australia

**Keywords:** numerosity perception, attention, segmentation, autistic quotient, grouping, connectedness

## Abstract

Perceptual grouping and visual attention are two mechanisms that help to segregate visual input into meaningful objects. Here we report how perceptual grouping, which affects perceived numerosity, is reduced when visual attention is engaged in a concurrent visual task. We asked participants to judge the numerosity of clouds of dot-pairs connected by thin lines, known to cause underestimation of numerosity, while simultaneously performing a color conjunction task. Diverting attention to the concomitant visual distractor significantly reduced the grouping-induced numerosity biases. Moreover, while the magnitude of the illusion under free viewing covaried strongly with AQ-defined autistic traits, under conditions of divided attention the relationship was much reduced. These results suggest that divided attention modulates the perceptual grouping of elements by connectedness and that it is independent of the perceptual style of participants.

## Introduction

To make sense of visual scenes, meaningful perception relies on our ability to quickly and efficiently organize visual information. The visual system groups elements using principles first introduced by Gestalt psychologists, including similarity, proximity, closure, and *common fate* (Wertheimer, 1923). This allows incoming information to be organized and integrated into coherent, whole objects, separate from the backgrounds. Selective attention is another process that influences how we perceive visual information. Attention and perceptual organization are interconnected, affecting visual processing and performance in various tasks and conditions.

Attentional demands in grouping have been investigated over the past decades, but the conclusions have been inconsistent (Ben-Av et al., 1992; Mack et al., 1992; Moore and Egeth, 1997; Kimchi and Razpurker-Apfeld, 2004; Lamy et al., 2006; see Kimchi, 2009, for a review). For example, when observers are engaged in an attentionally demanding task they are unable to report grouping organizations presented in the unattended backdrop of the task-relevant stimulus (Mack et al., 1992). Along these lines, apparent perceptual organization (luminance similarity) of a multielement array is intensified when attended and attenuated when unattended (Barbot et al., 2018), both suggesting that perceiving organization requires attention. In contrast, research using visual illusions whose susceptibility depends on grouping incoming information shows that individuals are susceptible to grouping even when they are unable to explicitly report the elements being grouped (Moore and Egeth, 1997; Driver et al., 2001; Russell and Driver, 2005; Lamy et al., 2006; Kimchi and Peterson, 2008; Shomstein et al., 2010; Carther-Krone et al., 2016). This view is further supported by studies involving patients with neurological disabilities such as hemispatial neglect (Russell and Driver, 2005; Shomstein et al., 2010) and simultanagnosia (Karnath et al., 2000; Huberle and Karnath, 2006), who can implicitly group elements despite difficulties explicitly reporting the global configuration.

In sum, the relationship between perceptual organization and attention is complicated: whereas some forms of perceptual organization can occur without attention (Braun and Sagi, 1991; Kimchi and Peterson, 2008), attention can nevertheless modulate perceptual organization processes.

In the present study, we used the numerosity illusion of *connectedness* to measure perceptual grouping. This illusion taps grouping mechanisms indirectly, without requiring participants to report directly the perceptual organization. When visual items such as circles or squares are grouped together with connecting lines, they appear less numerous (Franconeri et al., 2009; He et al., 2009; Anobile et al., 2017; Pomè et al., 2021a). The connecting lines are equally effective when very thin (Franconeri et al., 2009), or even when illusory (Kirjakovski and Matsumoto, 2016; Adriano et al., 2021). This has been taken as evidence that numerosity operates on segmented objects, defined by grouping, rather than individual local elements. For densely packed items, the effect of connectivity is greatly reduced (Anobile et al., 2017), showing that the effect is limited to the numerosity range of estimation of segregable items, rather than judgments of texture density. It also affects fMRI responses to numbers (He et al., 2015), adaptation to numbers (Fornaciai et al., 2016), and pupillometry (Castaldi et al., 2021).

Moreover, we recently demonstrated that the magnitude of the effect varies according to the perceptual styles of the participants: those scoring high on the self-reported Autistic Quotient questionnaire (AQ) showed a reduced illusory effect compared with participants with lower autistic traits. This is in line with theories that have linked autism with local processing and reduced awareness of the global aspects of stimuli (Pomè et al., 2021a).

The current work investigates whether grouping by connectedness can occur without attention being freely available to judge the numerosity of the stimulus. Recent evidence has shown that depriving visual attentional resources by a concomitant visual or auditory dual-task result in a higher cost in number representation in the small, *subitizing* number range than for larger numerosities (Burr et al., 2010; Pomè et al., 2019). Furthermore, the presentation of a visual cue that increases attentional engagement in a given task facilitates estimation, leading to a less compressive representation of numbers in space compared to when attention is diverted elsewhere (Pomè et al., 2021b). Numerosity benefits from object-based attentional resources, as cuing anywhere within an object gives the same attentional advantage as cuing the precise location of the object, suggesting that attention to number spreads from the cued location throughout the whole cued object (Pomè et al., 2021b).

**Figure 1:**
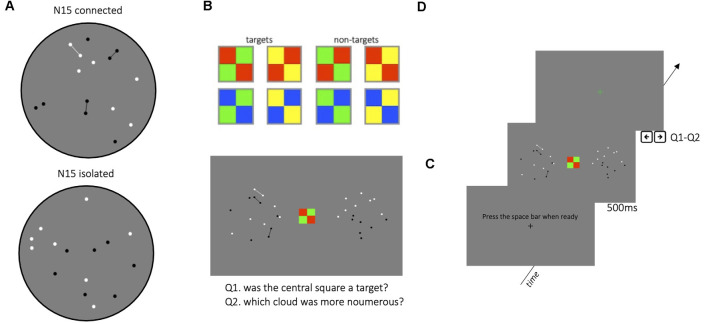
Illustration of the stimuli used in the experiment. **(A)** Example stimuli showing the connected condition (upper image), with 40% dots connected by thin lines, and the isolated-dot condition (lower image), with the lines, removed. Although both images have the same numerosity (N15), connecting dots with lines clearly causes the patch to appear less numerous. **(B)** Conjunction stimuli displayed in the center of the screen for the visual-spatial dual-task. The stimulus was a target if it satisfied a specific conjunction of colors and orientations (see the “Apparatus and Stimuli” section for details). **(C,D)** Example of the procedure with a timeline of a trial. Each trial started with a fixation point, followed by two- dot clouds presented together with the distractor, both for 500 ms. In the dual-task condition, participants responded first to the distractor task and then indicated which of the two clouds of dots seemed more numerous. In the single task, they performed only the numerosity task.

To investigate the dependence of grouping on attention, we measured the strength of the connectedness illusion (illustrated in Figure 1A) during divided attention. If grouping by connectivity is similarly strong it would suggest pre-attentive processing mechanisms responsible for perceptual grouping. On the other hand, if the illusion is reduced with divided attention, it would strongly implicate attention in implicit grouping processes.

## Materials and Methods

### Participants

Eighteen neurotypical young psychology students from the University of Florence participated in the experiment [11 females, age: 27.7 ± 2.7 (mean ± SD)]. All were naïve to the goals of the experiment but were experienced psychophysical observers who had all participated in previous psychophysical research. All had normal or corrected-to-normal visual acuity without major visual impairment. This sample size was deemed to be appropriate to obtain a moderate effect size with *α* = 0.05 and power of 0.8. All participants gave written informed consent, and experimental procedures were approved by the local ethics committee (“*Commissione per l’Etica della Ricerca*,” University of Florence, July 7, 2020, n. 111), and are in line with the declaration of Helsinki.

### AQ Scores

All participants completed the self-administered Autistic Quotient questionnaire, in either the validated Italian or English versions (Ruta et al., 2012; Ruzich et al., 2015). This contains 50 items, grouped in five subscales: attention switching, attention to detail, imagination, communication, and social skills. For each question, participants read a statement and selected the degree to which the statement best described them: “strongly agree,” “slightly agree,” “slightly disagree,” and “strongly disagree”. The standard scoring described in the original article was used: 1 when the participant’s response was characteristic of ASD (slightly or strongly), 0 otherwise. Total scores ranged between 0 and 50, with higher scores indicating higher degrees of autistic traits. All except one participant (with AQ 33) scored below 32, the threshold above which a clinical assessment is recommended (Baron-Cohen et al., 2001). The median of the scores was 13.5, with lower and upper quartiles of 8 and 22. Scores were normally distributed, as measured by the Jarque-Bera goodness-of-fit test of composite normality (JB = 1.14, *p* = 0.32).

### Apparatus and Stimuli

The experiment was run in a dimly lit room with stimuli presented on a 13-inch Macintosh monitor with resolution 2,560 × 1,600 pixels; refresh rate 60 Hz. Participants viewed the stimuli binocularly at a distance of 57 cm. The stimuli were generated and presented under Matlab 9.1 using PsychToolbox routines. Dots were small disks of 2.5 mm diameter (subtending 0.25° at 57 cm), half-white, half-black (so that luminance did not vary with a number, providing a potential cue). The stimuli for the numerosity task were two types of random dot-patterns, illustrated in Figure 1A: dots were either *isolated* (right image in Figure 1A), or with 40% of neighboring dots *connected* to create dumbbell-like shapes (left image). For patches containing isolated dots, dot positions were generated online to respect the sole condition that two items could not be closer than 2.5 mm (0.25°), preventing dot overlap. For the connected patterns, dot position was calculated in two stages: first couples of dots (40% of the total dots of the reference stimulus) were cast and connected *via* a line of the same color, with the constraints that line length was between 10 and 15 mm, with no lines crossing; in the second stage, the remaining 60% of dots were cast with the constraint of not overlapping either the other dots or the connecting lines. The connector line width was 0.5 mm.

Probe stimuli always comprised only *isolated* dots, but the constant-numerosity reference could comprise either isolated (baseline) or *connected* dots. In a particular session, one cloud of dots (the reference, randomly right or left) maintained a particular numerosity across trials, whereas the other (the probe) varied around this numerosity. The number of dots in the probe patch varied according to the QUEST adaptive algorithm (Watson and Pelli, 1983), perturbed with Gaussian noise with a standard deviation 0.15 log units. In separate blocks, three different reference numerosities were tested: 15, 25, 100. The dot stimuli were presented for 500 ms, simultaneously with a visual distractor. The visual distractors (Figure 1B) comprised four centrally positioned colored squares (3° × 3°), which could have eight color arrangements. The stimulus was a target if a specific conjunction of color and spatial arrangement was satisfied: two green squares along the right diagonal, or two yellow squares along the left diagonal.

### Procedure

In the single-task condition, participants indicated which of the two peripheral dot clouds contained more dots. In the dual-task condition, participants first responded to the distractor task and then indicated which of the two arrays was more numerous, using the right or left arrow on a keyboard (see Figures 1C,D). The order of tasks was pseudorandom across participants. Before starting the experimental condition, all participants were familiarized with the distractor task, in which they were asked to judge whether the central colored square was a target until they attained 75% accuracy; otherwise, the session was repeated. In the main experiment, all trials started with a fixation point presented until the participant pressed a key to start the experiment, and then the primary and secondary stimuli were presented for 500 ms. Participants were tested with three different reference numerosities. For each numerosity, they performed 180 trials, with the order of testing randomized across participants and conditions (connected or isolated), as well as the order of the tasks (single and dual). Participants were asked to maintain fixation on the central stimulus while performing both tasks. To ensure compliance, eye movements were monitored visually by the experimenter during all sessions. We verified that eye movements as small as 2° towards the peripheral stimuli (clouds of dots of 8 degree from central fixation) were readily observable. We reported no cases of observable eye movements under any condition, as may be expected for trained psychophysical observers.

### Data Analyses

For each participant, the proportion of trials in which the probe appeared more numerous than the reference was plotted against the number of reference dots and fit with a cumulative Gaussian error function. The median (the numerosity corresponding to 50% response) gave the point of subjective equality (PSE), and the difference in numerosity required to pass from 50% to 75% defined the JND, a measure of precision. The JND divided by the perceived numerosity yields the Weber Fraction (WF), a dimensionless index of precision that allows comparison of performance across numerosities. Our main analyses compared data across conditions (connected or isolated), tasks (single or dual), and groups of participants: ANOVAs and correlation analyses were complemented with Bayes factors estimation. Bayesian analyses were performed with the software JASP (entered with the per-condition, per-task, and per-subject averages computed in Matlab). Bayes Factors (Rouder et al., 2009) quantify the evidence for or against the null hypothesis as the ratio of the likelihoods for the experimental and the null hypothesis. We express it as the base 10 logarithm of the ratio (LogBF), where negative numbers indicate that the null hypothesis is likely to be true, positive that it is more likely false. By convention, *|*LogBF*|* > 0.5 is considered substantial evidence for either the alternate or null hypothesis, >1 strong evidence, and >2 decisive (van den Bergh et al., 2019).

## Results

**Figure 2:**
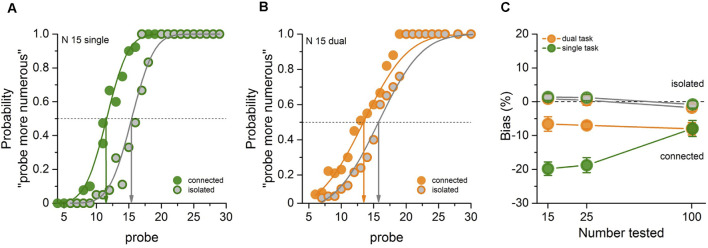
Psychometric functions for a typical participant for the single- **(A)** and dual-task **(B)** attentional condition at N15. The graphs plot the proportion of trials where the probe appeared more numerous than the reference (15 dots), as a function of probe numerosity (shown on the abscissa). The vertical arrows show the estimates of the point of subjective equality (PSE), given by the median of the fitted cumulative Gaussian functions. Green and orange respectively refer to single- and dual-task conditions; gray fillings refer to baseline (isolated) conditions, otherwise connected conditions. **(C)** Average PSEs as a function of dot number expressed as the percentage difference from the reference number, color-coded as in **(A,B)**, for both connected and isolated conditions in single- and dual-task.

We tested the effect of attentional load on perceptual grouping over a range of three different numerosities. Figures 2A,B show psychometric functions of an example participant for one numerosity (N15), for isolated and connected dots, in the two different attention conditions. For the single-task attentional condition (Figure 2A), there was a clear effect of connecting dots: when 40% of the reference dots were connected, the probe PSE was around 11 instead of 15, 27% less than the physical numerosity, agreeing with previous literature (Franconeri et al., 2009; He et al., 2009, 2015; Anobile et al., 2017). However, for the dual-task (Figure 2B), the shift was much reduced, only about two elements, or 13%. The point of subject equality (PSE) for the isolated dots in both single and dual was very near the physical numerosity of the reference (N15) in both cases, as to be expected.

Psychometric functions like those of Figures 2A,B were fit for each participant, condition and task, from which we extracted estimates of PSE for the various conditions. Figure 2C reports the average biases (expressed as percentage change) separately for the single and the dual (color-coded as in Figures 2A,B). For both tasks, the baseline biases (gray filled circles) were statistically indistinguishable from zero (*p* > 0.5), as to be expected. The bias of the connected stimuli for the single task was strong, and decreased with numerosity (mean ± SD: N15 = −19.81 ± 8.2; N25 = −18.87 ± 9.1, N100 = −7.91 ± 9.75) confirming our previous results (Anobile et al., 2017; Pomè et al., 2021a). However, the magnitude of the illusion was much less for the dual task condition at the lower numerosities (mean ± SD: N15 = −6.56 ± 8.8; N25 = −6.98 ± 4.2; N100 = −7.99 ± 7.43). This difference is revealed by the statistically significant main effects and interactions between numerosity and task for the connected condition. Two-way repeated measures ANOVA (two attentional conditions, three numerosities: Task *F*_(1,11)_ = 26.24, *p* < 0.001, logBF = 3.40, $\eta_{\text{p}}^{2}$ = 0.7; Numerosity *F*_(1,11)_ = 2.95, *p* = 0.07, logBF = 1.03, $\eta_{\text{p}}^{2}$ = 0.21; Task × Numerosity *F*_(2, 22)_ = 5.83; *p* = 0.009; LogBF = 1.56, $\eta_{\text{p}}^{2}$ = 0.34).

**Figure 3:**
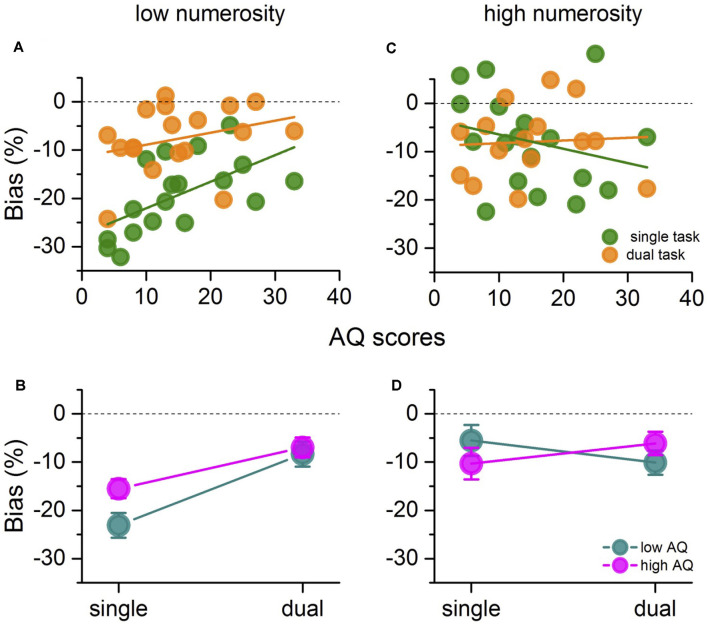
**(A)** Mean bias at low numerosities (N15-N25) plotted against AQ for all participants, for single- (green) and dual-task (orange). Thick green and orange lines show the linear fit of the data. **(B)** Mean underestimation bias for low (cyan) and high AQ (magenta), with error bars = ± 1 SEM, plotted as a function of the two tasks. **(C,D)** Same as in **(A)** and **(B)** but for high numerosity (N100).

As has been previously reported, when attention is not deprived, connecting dots of the low-density patterns reduces apparent numerosity considerably, while at higher densities the effect is less obvious. We, therefore, separated the data into low (N15–N25) and high (N100) numerosities to examine in more detail the relationship between biases and numerosities. As we were also interested in the effects of autistic personality traits especially on the condition of divided attention, we divided participants into low AQ (displayed as dark cyan) and high AQ (magenta), based on a median split of their AQ scores (above or below 13.5). Figure 3A plots the mean bias for the connected patch at low numerosities against AQ scores for both single and dual task. Results show a good correlation for the single task condition (*r* = 0.59; *p* < 0.05; logBF = 0.66): underestimation of the connected patches decreased with AQ scores. However, the dependency on AQ diminished and became insignificant for the dual task condition (*r* = 0.30; *p* = 0.2; logBF = −0.43). The Bayes factor is not strong (*|*LogBF*|* < 0.5), so it is not clear if the lack of significance results from there being no dependence, or lack of statistical power with the diminished effect. These differences are also evident in the mean underestimation effect for the low- and high-AQ groups shown in Figure 3B (mean ± SD: Single Task Low AQ = −23.08 ± 7.27; Single Task High AQ = −15.51 ± 5.9; Dual Task Low AQ = −8.32 ± 7.8; Dual Task high AQ = −6.95 ± 6.1). A two-way repeated measures ANOVA revealed main effect of task (*F*_(1,8)_ = 163.6, *p* < 0.001, logBF = 0.23, $\eta_{\text{p}}^{2}$ = 0.95), but no interaction between AQ and task (*F*_(1,8)_ = 2.5, *p* = 0.1, logBF = −0.39, $\eta_{\text{p}}^{2}$ = 0.24), and no main effect of AQ (*F*_(1,8)_ = 5.0, *p* = 0.057, logBF = −0.48, $\eta_{\text{p}}^{2}$ = 0.38) (although it is approaching significance, mainly driven by the differences in underestimation biases in the single task condition). Figure 3C shows the bias at high densities (N100). Here there is no correlation with AQ for either the single or the dual task (*r* = −0.25; *p* = 0.3; logBF = −0.53 for the single task and *r* = 0.06; *p* = 0.8; logBF = −0.70 for the dual task), and no significant difference between the average bias of the two groups in the two tasks (mean ± SD: Single Task Low AQ = −5.51 ± 9.6; Single Task High AQ = −10.31 ± 9.7; Dual Task Low AQ = −10.11 ± 7.5; Dual Task high AQ = −6.13 ± 7.31), as shown in Figure 3D (two way repeated measures ANOVA: main effect of task *F*_(1,6)_ = 0.032, *p* = 0.86, LogBF = −0.58, $\eta_{\text{p}}^{2}$ = 0.005); main effect of AQ *F*_(1,6)_ = 0.0006, *p* = 0.98, logBF = −0.56, $\eta_{\text{p}}^{2}$ = 0.0001; interaction *F*_(1,6)_ = 0.33, *p* = 0.58, logBF = −0.82, $\eta_{\text{p}}^{2}$ = 0.053). For both measures Bayes factors show substantial evidence for no effect.

**Figure 4:**
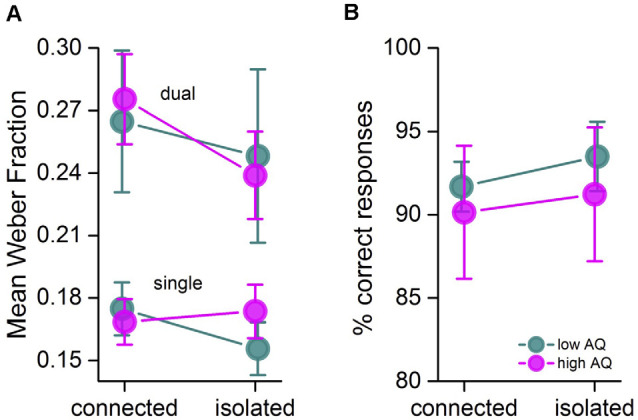
**(A)** Mean Weber fraction for discriminating numerosity in the isolated and connected conditions for the two groups and the two tasks. **(B)** Mean correct responses to the central visual distractor for the connected and isolated condition in the two groups. Color-coded as in Figure 3. Error bars = ± 1 SEM.

Figure 4A reports the Weber Fraction (WF) of the participants (averaged over all numerosities), given by the SD of the best-fitting Gaussians to the psychometric functions, normalized by the average perceived numerosity. Depriving attention clearly increased thresholds, by about 50%, in line with previous studies with peripheral stimuli (Pomè et al., 2019). However, the costs were similar for the isolated and connected conditions and for the high and low AQ groups (Condition *F*_(1,5)_ = 0.93, *p* = 0.38; logBF = −0.7, $\eta_{\text{p}}^{2}$ = 0.15; Task *F*_(1,5)_ = 10.3, *p* = 0.02 ; logBF = −1.55, $\eta_{\text{p}}^{2}$ = 0.67; AQ *F*_(1,5)_ = 0.07, *p* = 0.8; logBF = −0.77, $\eta_{\text{p}}^{2}$ = 0.01; Condition × task *F*_(1, 5)_ = 0.29, *p* = 0.62; logBF = −0.62, $\eta_{\text{p}}^{2}$ = 0.05; Condition × AQ *F*_(1,5)_ = 0.28, *p* = 0.62; logBF = −1.03, $\eta_{\text{p}}^{2}$ = 0.05; Task × AQ *F*_(1,5)_ = 0.01, *p* = 0.9; logBF = −0.68, $\eta_{\text{p}}^{2}$ = 0.003; Condition × Task × AQ *F*_(1, 5)_ = 20.5, *p* = 0.006; logBF = −1.39, $\eta_{\text{p}}^{2}$ = 0.80). And similar results were observed even when considering only the low numerosities, which could have affected the results also in terms of precision (Condition *F*_(1,4)_ = 0.11, *p* = 0.75; logBF = −0.74, $\eta_{\text{p}}^{2}$ = 0.02; Task *F*_(1,4)_ = 7.12, *p* = 0.056 ; logBF = 0.69, $\eta_{\text{p}}^{2}$ = 0.64; AQ *F*_(1,4)_ = 0.01, *p* = 0.9; logBF = −0.72, $\eta_{\text{p}}^{2}$ = 0.004; condition × task *F*_(1,4)_ = 0.27, *p* = 0.63; logBF = −0.62, $\eta_{\text{p}}^{2}$ = 0.06; condition × AQ *F*_(1,4)_ = 0.36, *p* = 0.57; logBF = −1.04, $\eta_{\text{p}}^{2}$ = 0.08; Task × AQ *F*_(1,4)_ = 0.59, *p* = 0.48; logBF = −0.56, $\eta_{\text{p}}^{2}$ = 0.013; Condition × Task × AQ *F*_(1,4)_ = 5.78, *p* = 0.07; logBF = −1.22, $\eta_{\text{p}}^{2}$ = 0.6).

We also calculated the percentage of correct responses to the central visual distractor (Figure 4B). Performance was very similar in both groups and conditions (excluding the possibility that the results were driven by deteriorated performance on the central distractor). Two-way repeated ANOVA confirmed that none of these effects were statistically significant (all *p* > 0.05, all $\eta_{\text{p}}^{2}$ < 0.2).

## Discussion

In this study, we examined the importance of visual attention for perceptual grouping in numerosity judgments. Participants judged which of two peripherally presented clouds of dots appeared more numerous, while simultaneously performing a difficult conjunction task. Apparent numerosity was manipulated by connecting dots with thin lines, known to cause underestimation of perceived numerosity, probably by grouping dot-pairs into objects. We replicated previous results (Franconeri et al., 2009; He et al., 2009; Anobile et al., 2017; Pomè et al., 2021a) when participants were free to attend to the dot-stimuli, with participants underestimating numerosity of the connected patch by around 20% for low-moderate numbers; however, the effect almost disappeared when attention was diverted to a concomitant attention-grabbing task, reduced to only 7%.

Together with principles first emphasized by Gestalt psychologists (Wertheimer, 1923) such as proximity, similarity, or common fate, uniform *connectedness* has been suggested as a grouping principle (Palmer and Rock, 1994): connecting a region of uniform visual properties causes it to be organized into a single perceptual unit. Several studies show that connecting dots with lines, as in this study, is a strong grouping facilitator, which dominates other factors, such as proximity and similarity. Connectedness can facilitate visual working memory, by organizing items into pre-packaged “chunks,” facilitating encoding of grouped items (Peterson and Berryhill, 2013). Connecting object parts has also been shown to influence the shift of visual attention (Watson and Kramer, 1999), multiple-object tracking (Scholl, 2001), and the performance of patients with visual neglect (Tipper and Behrmann, 1996). Studies have suggested that pairwise connecting of multiple targets significantly alters the spatial distribution of the attentional priority map, increasing the tendency of participants to jointly report or jointly miss elements that belonged to the same object (Gilchrist et al., 1997; Dent et al., 2011).

The present study provides further support that attention modulates the perceptual grouping of elements by connectedness. The results suggest that perceptual grouping affects numerosity estimation only after an attention-dependent grouping mechanism has generated a representation of a perceptual object. This implies that object completion requires sufficient attentional resources deployed to those parts of the visual field that could give rise to the perception of an integrated object; when the allocation of attention is prevented, such as by a concomitant attention-consuming visual task, this cannot proceed. From this perspective, attention may act like a “glue” to bind parts into wholes (Conci et al., 2018), contrary to the view that perceptual grouping may be considered “pre-attentive”. Future studies could examine the effects of enhancing attention instead of depriving it of numerosity grouping induced biases.

We have previously shown that the connectedness illusion is strongest for low to medium numerosity densities (Anobile et al., 2017), presumably because the items are less crowded and hence more segregable (Anobile et al., 2015, 2016; Burr et al., 2018). We replicate this effect here. Indeed, the effect of attention on the illusion at high densities was negligible, presumably because it was in any event much reduced.

One possible artifact is eye movements: in the single-task condition, participants could in theory have moved their eyes to foveate the targets, which may have made the thin grouping-lines more salient, whereas this would have been more difficult during the double-task condition. However, we believe this is most unlikely for several reasons. He et al. (2009) measured connected-induced grouping effects at various exposure durations, and observed, under similar eccentric conditions to ours, that the effect was strong (possibly stronger) at brief, 50-ms durations, too brief for foveation eye-movements to have occurred. Eye-movements are certainly not essential for the effect. In our experiment, while our participants were naïve to the goals of the experiment, they were trained psychophysical observers, well accustomed to maintaining fixation on instruction. To ensure compliance, the experimenter monitored eye-movements visually, after ascertaining that she could detect with perfect accuracy 2° deviations. As the targets were 8° eccentric, it would have been impossible for such a large movement to go unnoticed. We can therefore safely exclude this possibility.

We recently showed that perceiving the numerosity illusion is correlated with perceptual style: participants with higher self-reported autistic traits (AQ) are less susceptible to the connected numerosity illusion, suggesting that they are less susceptible to grouping effects. This is consistent with their having a more detail-oriented perceptual style, which has been linked with autism (Happe and Frith, 2006). In this study, the grouping advantage for low AQ participants disappeared under dual-task conditions. It is difficult to be certain whether the small remaining effect under deprived attention does not depend on AQ, or that the effect has simply become too small to reveal any dependence. The log10 Bayes factor for the correlation was −0.43, approaching −0.5 (the commonly accepted threshold for demonstrating a null effect), but we remain cautious in interpreting the results. However, we tentatively suggest that the lack of dependence of grouping on AQ when attention is diverted elsewhere is consistent with the same pattern of results for all participants, regardless of the perceptual style.

To conclude, the present study revealed that attention alters the perceived organization of multiple visual elements, furthering our understanding of the way attention modulates grouping by connectedness and impacts visual appearance. Overall, these findings advance our knowledge of the relationship between attention and perceptual organization, two prioritizing mechanisms that help to shape the way we experience our visual world.

## Data Availability Statement

The raw data supporting the conclusions of this article will be made available by the authors, without undue reservation, under reasonable request.

## Ethics Statement

The studies involving human participants were reviewed and approved by Commissione per l’Etica della Ricerca, università di Firenze. The patients/participants provided their written informed consent to participate in this study.

## Author Contributions

All authors contributed to the study concept and to the design. Stimuli were designed by AP and CC. Testing and data collection and data analysis were performed by AP and CC. All authors contributed to the interpretation of the results. AP drafted the manuscript and DB provided critical revisions. All authors contributed to the article and approved the submitted version.

## Conflict of Interest

The authors declare that the research was conducted in the absence of any commercial or financial relationships that could be construed as a potential conflict of interest.

## Publisher’s Note

All claims expressed in this article are solely those of the authors and do not necessarily represent those of their affiliated organizations, or those of the publisher, the editors and the reviewers. Any product that may be evaluated in this article, or claim that may be made by its manufacturer, is not guaranteed or endorsed by the publisher.

## References

[B1] Adriano A. Rinaldi L. Girelli L. (2021). Visual illusions as a tool to hijack numerical perception: disentangling nonsymbolic number from its continuous visual properties. J. Exp. Psychol. Hum. Percept. Perform. 47, 423–441. 10.1037/xhp0000844 33492161

[B2] Anobile G. Cicchini G. M. Burr D. C. (2016). Number as a primary perceptual attribute: a review. Perception 45, 5–31. 10.1177/0301006615602599 26562858 PMC5040510

[B3] Anobile G. Cicchini G. M. Pomè A. Burr D. C. (2017). Connecting visual objects reduces perceived numerosity and density for sparse but not dense patterns. J. Num. Cogn. 3, 133–146. 10.5964/jnc.v3i2.38

[B4] Anobile G. Turi M. Cicchini G. M. Burr D. C. (2015). Mechanisms for perception of numerosity or texture-density are governed by crowding-like effects. J. Vis. 15:4. 10.1167/15.5.4 26067522 PMC4909146

[B6] Barbot A. Liu S. Kimchi R. Carrasco M. (2018). Attention enhances apparent perceptual organization. Psychon. Bull. Rev. 25, 1824–1832. 10.3758/s13423-017-1365-x 28849553 PMC5831484

[B7] Baron-Cohen S. Wheelwright S. Skinner R. Martin J. Clubley E. (2001). The autism-spectrum quotient (AQ): evidence from asperger syndrome/high-functioning autism, males and females, scientists and mathematicians. J. Autism Dev. Disord. 31, 5–17. 10.1023/a:1005653411471 11439754

[B8] Ben-Av M. B. Sagi D. Braun J. (1992). Visual attention and perceptual grouping. Percept. Psychophys. 52, 277–294. 10.3758/bf03209145 1408639

[B9] Braun J. Sagi D. (1991). Texture-based tasks are little affected by second tasks requiring peripheral or central attentive fixation. Perception 20, 483–500. 10.1068/p200483 1771133

[B10] Burr D. C. Anobile G. Arrighi R. (2018). Psychophysical evidence for the number sense. Philos. Trans. R. Soc. Lond. B Biol. Sci. 373:20170045. 10.1098/rstb.2017.0045 29292350 PMC5784049

[B11] Burr D. C. Turi M. Anobile G. (2010). Subitizing but not estimation of numerosity requires attentional resources. J. Vis. 10:20. 10.1167/10.6.20 20884569

[B12] Carther-Krone T. A. Shomstein S. Marotta J. J. (2016). Looking without perceiving: impaired preattentive perceptual grouping in autism spectrum disorder. PLos One 11:e0158566. 10.1371/journal.pone.0158566 27355678 PMC4927180

[B13] Castaldi E. Pome A. Cicchini G. M. Burr D. C. Binda P. (2021). Pupil size automatically encodes numerosity. J. Vis. 21:2302. 10.1167/jov.21.9.2302

[B14] Conci M. Groß J. Keller I. Müller H. J. Finke K. (2018). Attention as the ‘glue’ for object integration in parietal extinction. Cortex 101, 60–72. 10.1016/j.cortex.2017.12.024 29454223

[B15] Dent K. Humphreys G. W. Braithwaite J. J. (2011). Spreading suppression and the guidance of search by movement: evidence from negative color carry-over effects. Psychon. Bull. Rev. 18, 690–696. 10.3758/s13423-011-0091-z 21484507

[B16] Driver J. Davis G. Russell C. Turatto M. Freeman E. (2001). Segmentation, attention and phenomenal visual objects. Cognition 80, 61–95. 10.1016/s0010-0277(00)00151-7 11245840

[B17] Fornaciai M. Cicchini G. M. Burr D. C. (2016). Adaptation to number operates on perceived rather than physical numerosity. Cognition 151, 63–67. 10.1016/j.cognition.2016.03.006 26986745 PMC5040501

[B18] Franconeri S. L. Bemis D. K. Alvarez G. A. (2009). Number estimation relies on a set of segmented objects. Cognition 113, 1–13. 10.1016/j.cognition.2009.07.002 19647817

[B19] Gilchrist I. D. Humphreys G. W. Riddoch M. J. Neumann H. (1997). Luminance and edge information in grouping: a study using visual search. J. Exp. Psychol. Hum. Percept. Perform 23, 464–480. 10.1037//0096-1523.23.2.464 9104005

[B20] Happe F. Frith U. (2006). The weak coherence account: detail-focused cognitive style in autism spectrum disorders. J. Autism Dev. Disord. 36, 5–25. 10.1007/s10803-005-0039-0 16450045

[B21] He L. Zhang J. Zhou T. Chen L. (2009). Connectedness affects dot numerosity judgment: implications for configural processing. Psychon. Bull Rev. 16, 509–517. 10.3758/PBR.16.3.509 19451377

[B22] He L. Zhou K. Zhou T. He S. Chen L. (2015). Topology-defined units in numerosity perception. Proc. Natl. Acad. Sci. U S A 112, E5647–E5655. 10.1073/pnas.1512408112 26417075 PMC4611617

[B23] Huberle E. Karnath H.-O. (2006). Global shape recognition is modulated by the spatial distance of local elements—Evidence from simultanagnosia. Neuropsychologia 44, 905–911. 10.1016/j.neuropsychologia.2005.08.013 16226773

[B24] Karnath H.-O. Ferber S. Rorden C. Driver J. (2000). The fate of global information in dorsal simultanagnosia. Neurocase 6, 295–306. 10.1080/13554790008402778

[B25] Kimchi R. (2009). Perceptual organization and visual attention. Prog. Brain Res. 176, 15–33. 10.1016/S0079-6123(09)17602-1 19733747

[B27] Kimchi R. Peterson M. A. (2008). Figure-ground segmentation can occur without attention. Psychol. Sci. 19, 660–668. 10.1111/j.1467-9280.2008.02140.x 18727781

[B26] Kimchi R. Razpurker-Apfeld I. (2004). Perceptual grouping and attention: not all groupings are equal. Psychon. Bull. Rev. 11, 687–696. 10.3758/bf03196621 15581119

[B28] Kirjakovski A. Matsumoto E. (2016). Numerosity underestimation in sets with illusory contours. Vis. Res. 122, 34–42. 10.1016/j.visres.2016.03.005 27038561

[B29] Lamy D. Segal H. Ruderman L. (2006). Grouping does not require attention. Percept. Psychophys. 68, 17–31. 10.3758/BF03193652 16617826

[B30] Mack A. Tang B. Tuma R. Kahn S. Rock I. (1992). Perceptual organization and attention. Cogn. Psychol. 24, 475–501. 10.1016/0010-0285(92)90016-u 1473332

[B31] Moore C. M. Egeth H. (1997). Perception without attention: evidence of grouping under conditions of inattention. J. Exp. Psychol. Hum. Percept. Perform. 23, 339–352. 10.1037//0096-1523.23.2.339 9103998

[B32] Palmer S. Rock I. (1994). Rethinking perceptual organization: the role of uniform connectedness. Psychon. Bull. Rev. 1, 29–55. 10.3758/BF03200760 24203413

[B33] Peterson D. J. Berryhill M. E. (2013). The gestalt principle of similarity benefits visual working memory. Psychon. Bull. Rev. 20, 1282–1289. 10.3758/s13423-013-0460-x 23702981 PMC3806891

[B36] Pomè A. Anobile G. Cicchini G. M. Scabia A. Burr D. C. (2019). Higher attentional costs for numerosity estimation at high densities. Atten. Percept. Psychophys. 81, 2604–2611. 10.3758/s13414-019-01831-3 31407272 PMC6856040

[B34] Pomè A. Caponi C. Burr D. C. (2021a). Grouping-induced numerosity biases vary with autistic-like personality traits. J. Autism Dev. Disord. 10.1007/s10803-021-05029-1 [Online ahead of print]. 33909210 PMC8854316

[B35] Pomè A. Thompson D. Burr D. C. Halberda J. (2021b). Location- and object-based attention enhance number estimation. Atten. Percept. Psychophys. 83, 7–17. 10.3758/s13414-020-02178-w 33156512 PMC7875840

[B37] Rouder J. N. Speckman P. L. Sun D. Morey R. D. Iverson G. (2009). Bayesian t tests for accepting and rejecting the null hypothesis. Psychon. Bull. Rev. 16, 225–237. 10.3758/PBR.16.2.225 19293088

[B38] Russell C. Driver J. (2005). New indirect measures of “inattentive” visual grouping in a change-detection task. Percept. Psychophys. 67, 606–623. 10.3758/bf03193518 16134455

[B39] Ruta L. Mazzone D. Mazzone L. Wheelwright S. Baron-Cohen S. (2012). The autism-spectrum quotient–italian version: a cross-cultural confirmation of the broader autism phenotype. J. Autism Dev. Disord. 42, 625–633. 10.1007/s10803-011-1290-1 21626054

[B40] Ruzich E. Allison C. Smith P. Watson P. Auyeung B. Ring H.. (2015). Measuring autistic traits in the general population: a systematic review of the Autism-spectrum Quotient (AQ) in a nonclinical population sample of 6,900 typical adult males and females. Mol. Autism 6:2. 10.1186/2040-2392-6-2 25874074 PMC4396128

[B41] Scholl B. J. (2001). Objects and attention: the state of the art. Cognition 80, 1–46. 10.1016/s0010-0277(00)00152-9 11245838

[B42] Shomstein S. Kimchi R. Hammer M. Behrmann M. (2010). Perceptual grouping operates independently of attentional selection: evidence from hemispatial neglect. Atten. Percept. Psychophys. 72, 607–618. 10.3758/APP.72.3.607 20348567 PMC2893411

[B43] Tipper S. P. Behrmann M. (1996). Object-centered not scene-based visual neglect. J. Exp. Psycho. Hum. Percept. Perform. 22, 1261–1278. 10.1037//0096-1523.22.5.1261 8865621

[B400] van den Bergh D. van Doorn J. Marsman M. Draws T. van Kesteren E. Derks K.. (2019). A tutorial on conducting and interpreting a bayesian ANOVA in JASP. L’Année Psychologique 120, 73–96. 10.3917/anpsy1.201.007325874074

[B45] Watson S. E. Kramer A. F. (1999). Object-based visual selective attention and perceptual organization. Percept. Psychophys. 61, 31–49. 10.3758/bf03211947 10070198

[B44] Watson A. B. Pelli D. G. (1983). QUEST: a bayesian adaptive psychometric method. Percept. Psychophys. 33, 113–120. 10.3758/bf03202828 6844102

[B46] Wertheimer M. (1923). Untersuchungen zur Lehre von der Gestalt. II. Psychol. Forsch. 4, 301–350. 10.1007/BF00410640

